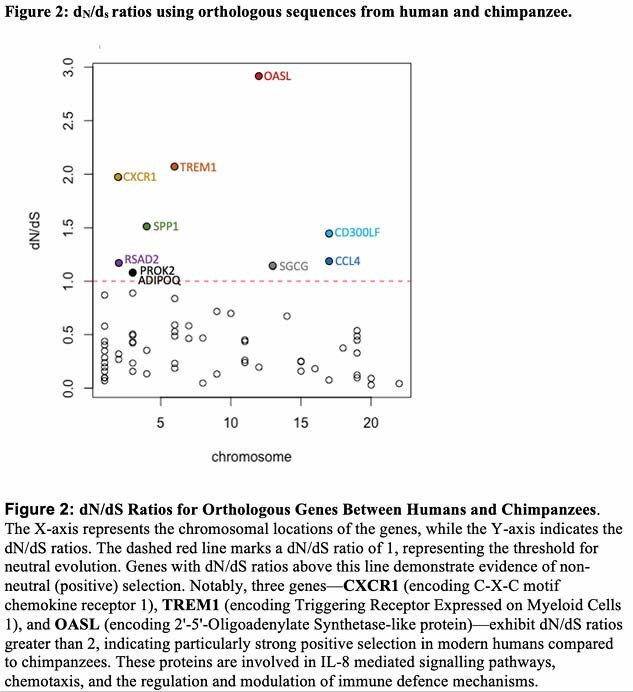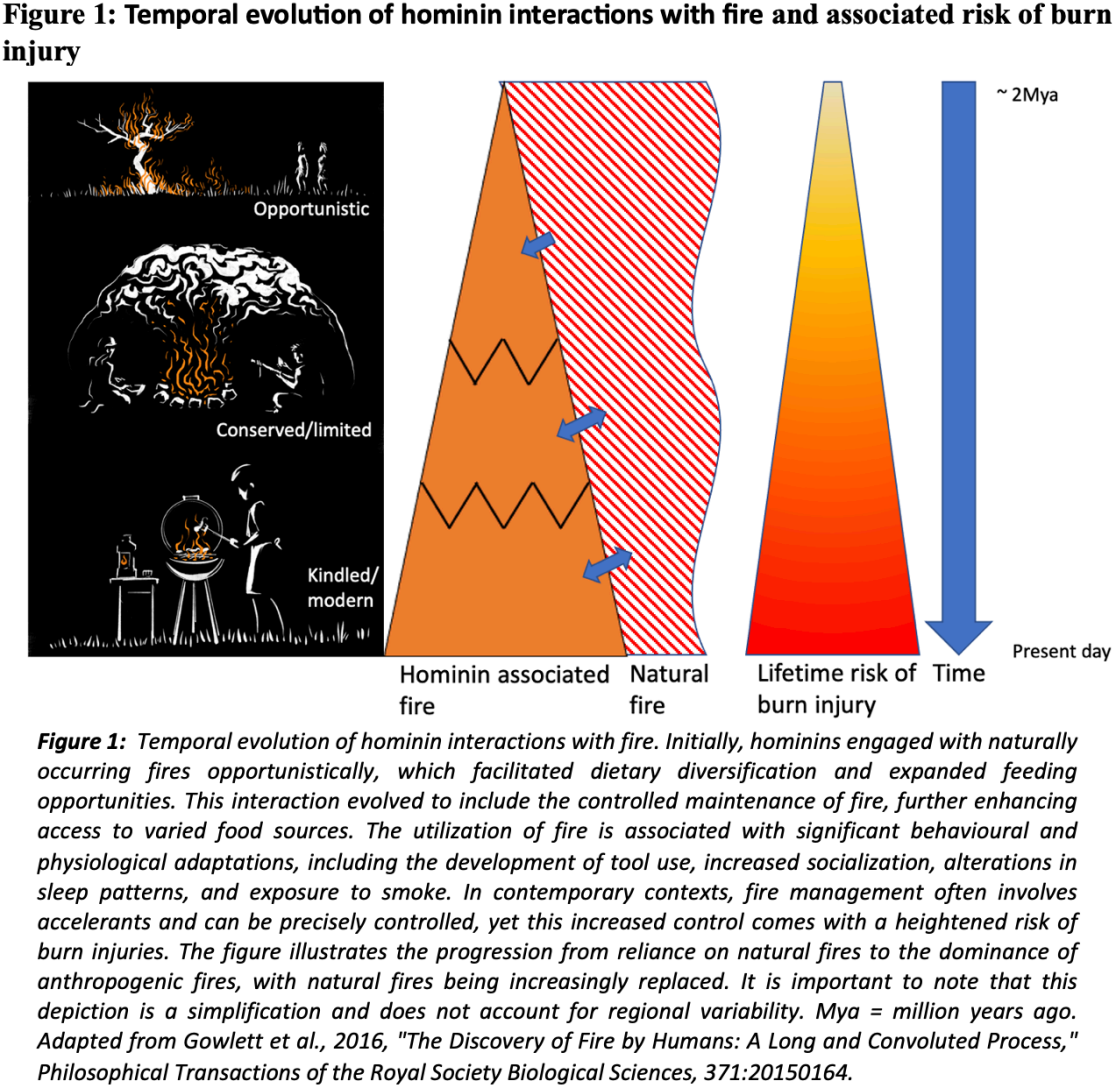# 859 The Selection Through Burn Injury Hypothesis: How Burn Injuries Shaped Human Evolution

**DOI:** 10.1093/jbcr/iraf019.390

**Published:** 2025-04-01

**Authors:** Josh Cuddihy, Yuemin Li, Matteo Fumagalli, Marcela Vizcaychipi, Declan Collins, Dominic Friston, Istvan Nagy

**Affiliations:** Chelsea and Westminster Hospital NHS Foundation Trust; Queen Mary university London; Queen Mary university London; Imperial College London; Chelsea and Westminster Hospital, Imperial College London; Imperial College London; Imperial College London

## Abstract

**Introduction:**

The mastery of fire was crucial in human evolution but likely increased the risk of skin burn injuries in hominins more than other species. Our “selection through burn injury hypothesis” suggests that these injuries became a significant evolutionary selective pressure throughout human development.

**Methods:**

To explore the genetic implications, we compared the post-burn and non-burned transcriptomes of rats and humans using publicly available microarray data. Matched human and rat ortholog pairs (21,597 genes) were analyzed to identify differentially expressed human genes (DEGs) with a log fold change of > 1.5 or < -1.5, resulting in 94 DEGs. These DEGs were compared using dN/dS analysis between publicly available orthologous human and chimpanzee sequences to find evidence of non-neutral selection. Population genetic analysis (Tajima’s D and population branch statistics) was performed on the same DEGs using genomic data from 108 Yoruba (YRI) from Ibadan, Nigeria, 99 individuals of Northern and Western European ancestry (CEU), and 103 Han Chinese in Beijing (CHB), representing African, European, and Asian populations, respectively. GO terms analysis was conducted on the identified genes showing evidence of non-neutral selection.

**Results:**

Ten of the DEGs genes showed dN/ds evidence of positive selection more prevalent in human compared to chimpanzee populations, greater than that expected by chance alone. Further there was evidence of non-neutral selection in DEGs between modern human populations, greater than expected by chance alone. GO terms analysis. GO-slim biological processes analysis showed Neutrophil, granulocyte, myeloid leukocyte and leukocyte migration biological processes linked strongly to the genes analysed as did inflammatory cell chemotaxis processes, with response to external biotic stimulus and defence responses.

**Conclusions:**

Our analysis provides evidence for positive selection of a series of genes involved in burn injury wound healing in human burn injury, greater than that expected by chance alone, in support of our novel theory of selection through burn injuries. Specific genes identified are implicated with pathological processes involved in burn healing, including upregulated inflammation and wound closure, scar tissue formation and nociception.

**Applicability of Research to Practice:**

We propose that this understanding provides valuable insight into the pathological processes following burn injury and how these responses may have provided a survival advantage despite seemingly causing complication in burn injured patients. Additionally, the evolving selective pressures on immune systems evolved to principally combat pathogen invasion may explain the initial apparent dysregulated immune responses in humans following tissue loss from a sterile burn injury. This new theory may change perspectives of human evolution and directs research into developing therapies for burn injury.

**Funding for the Study:**

N/A